# Comparing COVID-19-linked neurological complications with other viral infections

**DOI:** 10.1186/s12967-020-02633-0

**Published:** 2020-12-09

**Authors:** Xiao Deng, Yew-Long Lo, Eng -King Tan

**Affiliations:** Department of Neurology, National Neuroscience Institute, Duke NUS Medical School, Singapore General Hospital, Outram Road, Singapore, 169608 Singapore

**Keywords:** COVID-19, SARS, Influenza, Cerebrovascular disease

There are increasing reports of multi-system involvement in coronavirus disease 2019 (COVID-19). Among these, cerebrovascular diseases (CVD) in COVID patients have been highlighted to be associated with varied features and prognosis. COVID-19 may possess the similar mechanism and clinical characteristics as SARS, as the viruses causing them are under the same category and share highly homological genetic sequence. Influenza H1N1 and COVID-19 are comparable in terms of outbreak size as Influenza H1N1 also bring about global pandemic. However, to date, a detailed systematic comparison of neurological complications among COVID-19, SARS-CoV-1 and Influenza H1N1 infections has not been carried out.

To address this gap in knowledge, we compared the frequency, presentation and prognosis of neurological complications in COVID-19 with SARS and Influenza H1N1 infection (Tables 1, 2). This may provide further mechanistic insights into potential differences between COVID-19 and other viral infections.

**Table 1:** Severe neurological symptoms among different viral infections

Neurologic disorders	COVID-19 SARS-CoV-2 infection 2019	SARS SARS-CoV-1 infection 2003	INFLUENZA H1N1 infection 2009
Frequency	2.8% (6/214) patients with acute cerebrovascular disease, Wuhan China [1] 13.8% (8/58) required intensive care, France [13] Globally, 93 patients with encephalopathy; 19 patients with Guillain-Barré syndrome; 8 cases with encephalitis have been reported [14]	2.4% (5/206) with large-vessel stroke, Singapore [2] 5.3% (4/76) with neuromuscular symptoms, Taiwan [5]	9.1% (5/55) with severe neurological symptoms, Iran [4]
Onset of Neurologic manifestations	Can be both early and late onset of neurological manifestations [3]	Two to three weeks after the onset of SARS [2, 5]	Within 7 days
Common neurological manifestations in severe cases	Impaired consciousness; Acute cerebrovascular disease, Skeletal muscle injury	Polyneuropathy, encephalitis, and aortic ischemic stroke	Seizures, encephalopathy and encephalitis
Prognosis	More patients have neurologic disorders in severe subtype with poor outcome	Poor	More children than adults were identified to have neurologic injury with poor outcome [7]
Possible mechanism	The neuroinvasive potential of SARS‐CoV2 may play a role in the respiratory failure [11]	ACE2 [11] and immune injury may play a role	Direct infection, hypoxia and metabolite dysfunction may be more significant [12]

**Table 2:** Cerebrovascular disease among different COVID-19 reports

Acute cerebrovascular disease	New York USA case report (n = 5) [9]	London UK case report (n = 6) [3]	Philadelphia USA Case report (n = 2) [15]	Wuhan China case series (n = 6) [1]
Onset age	Younger than 50 years (ranged from 33 to 49); 4 of them were male	Ranged from 53–85 years old; 5 of them were male	Ranged from 31 and 62 years old; 1 of them were male	Patients with severe infection were older (58.2 ± 15.0)and had more acute CVD
Stroke type	Large-vessel ischemic stroke	All had large vessel occlusion with markedly elevated D-dimer levels. 3 had multi-territory infarcts; 2 had concurrent venous thrombosi	One is subarachnoid haemorrhage from a ruptured aneurysm; another is ischaemic stroke with massive haemorrhagic conversion	Five patients with ischemic stroke and 1 with cerebral hemorrhage
Comorbidity	One patient had history of stroke; 2 had diabetes; 1 had hyperlipidemia	Majority 5/6 had multiple comorbidities including cardiopathy, hypertension, diabetes, cancer and previous stroke history	No	Patients with severe infection had more underlying disorders, especially hypertension, and showed fewer typical symptoms of COVID-19, such as fever and cough
Prognosis	One out of five was sent to intensive care unit	Two out of 6 required intensive care unit support	Zero out of two required intensive care unit	One out of six deceased
Potential mechanism	Possible due to Coagulopathy and vascular endothelial dysfunction	Coagulation activation and thrombin generation due to proinflammatory cytokines which induce endothelial and mononuclear cell activation	Underlying inflammatory and hypercoagulable state may incite cerebrovascular disease without disruption of the blood–brain barrier	ACE2 and immune injury may play a role

We searched PubMed from January, 2020 to June, 2020. The following key words were included: “COVID-19”, “SARS”, “influenza”, “cerebrovascular disease”, “neurological symptoms”, “neurological manifestations”. Two review authors (EK-T, XD) independently reviewed the included studies and extracted study characteristics.

About 3% of COVID-19 patients reported acute CVD in a study from Wuhan [1], comparable to the reported frequency of 2.42% for SARS reported in Singapore [2]. Neurologic manifestations occurred both early and late stage in the course of the COVID-19 [3], with more clinical variability than SARS and H1N1 infections. The most common severe neurologic manifestations in COVID-19 patients included acute CVD, impaired consciousness, and skeletal muscle injury, which appeared slightly higher than SARS and H1N1 patients [1, 2, 4, 5]. Acute symptomatic seizures or status epilepticus were not seen commonly in COVID-19 patients [6]. More children with H1N1 than adults suffered neurologic injury with poor outcome [7], whereas paediatric patients with COVID-19 were more likely to have better outcome than adults [8] (Table 1).

For CVD in COVID-19, we noticed that a number of them were relatively young (less than 50 years and of male gender) (Table 2). There was no consistent pattern to the types of strokes, with reports of involvement in small, medium or large vessels [3, 9] (Table 2). The blockages of these vessels led to infarcts and in some cases frank bleeding. The prognosis depended on the severity of the strokes at presentations and associated complications. Not surprisingly, vascular risk factors such as hyperlipidaemia, diabetes and hypertension were present especially in the older group of patients [10]. These risk factors were not different from the common stroke patients seen during non-COVID-19 period. Several patients in one series [3] have been reported to have a positive lupus anticoagulant, which may have predisposed them to the disease. However, it is not clear if these patients were more susceptible to COVID-19 or if there was a complex interplay of the factors involved. It is possible that proinflammatory cytokines contributed to the blockage of the vessels [3]. For those stroke patients who were disabled, long term data on the final recovery outcomes were still not available.

The infection of SARS‐CoV can affect brains, especially the brainstem mainly mediated by a cellular receptor angiotensin‐converting enzyme 2 (ACE2) [11], which can be expressed in human airway epithelia, lung parenchyma, vascular endothelia. The similarity of severe neurological manifestations in COVID-19 and in SARS patients indicate that ACE2 may also play a role in the underlying mechanism. In addition, the respiratory failure in COVID-19 patients may result from the neuroinvasive potential of SARS‐CoV2 [11]. Different from the possible mechanisms of SARS-CoV-1 and SARS-CoV-2 infections, H1N1 infection might be due to direct infection, hypoxia and metabolite dysfunction [12].

To summarise, based on current data, the frequency of CVD in COVID-19 appeared slightly more than SARS and H1N1 patients. COVID-19 had more variability than SARS and H1N1 patients in terms of the onset of neurologic manifestations. Longitudinal studies to further clarify the chronic neurological burden could be particularly useful to stratify COVID-19 patients and guide the medical recourse allocation. It could be particularly useful to guide strategic planning for current and future pandemics. Functional studies to decipher the pathophysiologic mechanism, in particular the role of the COVID-19 in vessel wall inflammation, blockage and secondary cytokine response will be warranted.

## Data Availability

Not applicable.

## Abbreviations

COVID-19: Coronavirus disease 2019 infection
SARS: Severe Acute Respiratory Syndrome
H1N1: Influenza A virus subtype H1N1
CVD: Cerebrovascular diseases
